# Genetic and Transcriptomic Biomarkers in Neurodegenerative Diseases: Current Situation and the Road Ahead

**DOI:** 10.3390/cells10051030

**Published:** 2021-04-27

**Authors:** Julie Lake, Catherine S. Storm, Mary B. Makarious, Sara Bandres-Ciga

**Affiliations:** 1 Laboratory of Neurogenetics, National Institute on Aging, National Institutes of Health, Bethesda, MD 20892, USA; julianne.lake@nih.gov (J.L.); mary.makarious@nih.gov (M.B.M.); 2 Department of Clinical and Movement Neurosciences, UCL Queen Square Institute of Neurology, London WC1N 3BG, UK; catherine.storm.14@ucl.ac.uk; 3 UCL Movement Disorders Centre, University College London, London WC1E 6BT, UK

**Keywords:** Alzheimer’s disease, Parkinson’s disease, amyotrophic lateral sclerosis, biomarkers, genetics, transcriptomics

## Abstract

Neurodegenerative diseases are etiologically and clinically heterogeneous conditions, often reflecting a spectrum of disease rather than well-defined disorders. The underlying molecular complexity of these diseases has made the discovery and validation of useful biomarkers challenging. The search of characteristic genetic and transcriptomic indicators for preclinical disease diagnosis, prognosis, or subtyping is an area of ongoing effort and interest. The next generation of biomarker studies holds promise by implementing meaningful longitudinal and multi-modal approaches in large scale biobank and healthcare system scale datasets. This work will only be possible in an open science framework. This review summarizes the current state of genetic and transcriptomic biomarkers in Parkinson’s disease, Alzheimer’s disease, and amyotrophic lateral sclerosis, providing a comprehensive landscape of recent literature and future directions.

## 1. Introduction

In 1998, the National Institutes of Health’s Biomarkers Definitions Working Group defined a biomarker as “a characteristic that is objectively measured and evaluated as an indicator of normal biological processes, pathogenic processes, or pharmacologic responses to a therapeutic intervention.” [1]. It is widely assumed that a successful biomarker must be objective, inexpensive, accessible, accurate in a diverse group of individuals and easily quantifiable, and correlate with the presence or severity of the disease [2].

In the neurodegenerative diseases field, the discovery and validation of biomarkers is an area of ongoing effort and interest. A plethora of studies have been conducted in an attempt to unravel biomarkers that may be characteristic indicators for preclinical disease diagnosis (before clinical symptoms occur), predictive prognosis, and disease subtyping. In this arena, the search may be particularly difficult because these conditions are not clearly defined entities. They are etiologically and clinically heterogeneous, and they may rather reflect a spectrum of neurodegenerative disease processing. The intra- and inter-patient variation and the fact that co-pathologies are frequent and have complex contributions to clinical phenotypes makes biomarker discovery particularly challenging.

Over the years, biomarker studies conducted in the field of neurogenetics have usually focused on identifying single biomarker metrics with limited applicability (Table 1)**.**

These genetic markers are often disease-causing deleterious mutations responsible for monogenic forms of disease. However, even in the majority of the cases, the relationship between a genetic biomarker and the development of the disease is complex, due to the variability of penetrance and the contribution of genetic risk factors interplaying with the environment.

The underlying molecular complexity in neurodegenerative diseases has made the next generation of biomarker studies take shape as meaningful multi-modal approaches using large scale biobank datasets. To a large extent, our current knowledge about the etiology underlying neurodegenerative diseases has been driven by advances in the known ”-omics”, including genomics, transcriptomics, proteomics, and metabolomics. Despite being widely applied in research, the road towards a successful implementation and translation into the clinic is in its early stages. The availability of reliable biomarkers able to provide an early diagnosis and the identification of individuals at risk, monitor disease progression and allow the discovery of novel and more individualized treatments for these debilitating conditions is urgently needed in our search for a cure.

This review aims at providing a general overview on the current status of genetic and transcriptomic biomarkers in the era of big data and precision medicine by focusing on the most common neurodegenerative diseases, including Parkinson’s disease (PD), Alzheimer’s disease (AD), and amyotrophic lateral sclerosis (ALS). We assess the progress achieved so far and discuss the main challenges and limitations in our way to dissect the complexity underlying these debilitating conditions.

## 2. Parkinson’s Disease

Parkinson’s disease (PD) is a neurodegenerative movement disorder in which the diagnosis is currently based on the patient’s clinical history and examination. The clinical diagnosis at first visit is, however, only accurate in 80% of pathologically-confirmed PD [3]. The classical presentation includes progressive slow movements, resting tremor, and stiffness [4], and patients often report long-standing, prodromal non-motor symptoms [4]. Dopamine transporter imaging can be helpful for diagnosis when the examination does not clearly reveal parkinsonism, but its usefulness is limited when parkinsonian motor signs are unequivocally present [4]. There has been extensive research searching for protein biomarkers in cerebrospinal fluid and blood [5], but these findings have not yet been translated to the clinic. As such, there remains an unmet need for objective biomarkers for early-stage diagnosis [6].

### 2.1. Genetic Biomarkers

#### 2.1.1. Rare Mutations

While the cause of PD is unclear, there are several genetic and environmental risk factors. The genetic contributors to PD risk lie on a spectrum from rare variants with strong effects to common variants with weak effects. A minority of PD cases carry rare mutations that are sufficient to cause a familial or monogenic form of neurodegeneration, reviewed in references [7,8,9]. These include mutations and/or copy number variants in *SNCA, LRRK2, PINK1, PARK2, DJ1,* or *VPS35*. While these mutations can be considered relatively reliable biomarkers for some patients, the vast majority of PD cases do not have a clear genetic cause. As such, genetic variation is usually considered a risk factor for this disease. For example, mutations in the *GBA* gene have been linked to roughly a fivefold increase in PD risk [10]. As previously mentioned, the clinical usefulness of these mutations is limited by their low prevalence and incomplete penetrance [11,12].

#### 2.1.2. Common Variants and Polygenic Risk Scores

Genome-wide association studies (GWASs) have identified over 90 common genetic variants associated with PD risk in Europeans, and 11 in Asian populations [13,14]. While each GWAS-identified variant accounts for a very small proportion of this risk, variants can be aggregated to form a polygenic risk score (PRS). Using the effect sizes and alleles calculated for each variant in the GWAS, a PRS could be used as a biomarker to estimate an individual’s risk of disease. Several studies have shown that GWAS-derived PRSs correlate with disease risk, age at onset, as well as motor and cognitive decline (measured by change in UPDRS part III score, time to Hoehn and Yahr stage 3, change in the mini-mental state examination), but not survival [15,16,17,18,19].

Nevertheless, genetic testing does not currently have an established role in the diagnostic process unless the patient’s history prompts suspicion for a genetic cause through, for example, a family history or early motor symptom onset. Calculating an individual’s PRS would need to have a substantial impact on clinical trial recruitment or patient quality of life before it could be implemented. Genetic variation is estimated to account for about 22% of PD risk, and to date only 16–36% of that risk may be explained by GWAS-identified loci (depending on the estimated disease prevalence) [13]. It is thus unlikely that such a PRS alone could currently have a substantial impact on patient care. Furthermore, the vast majority of GWAS data is based on individuals of European descent only. The less an individual is genetically similar to the GWAS study population, the less accurate the PRS will be in predicting disease risk in that individual [20,21,22]. As such, current PRSs do not yet reach the diagnostic accuracy needed to be translated to the clinic.

### 2.2. Transcriptomic Biomarkers

Beyond genetics, the potential of RNA-based biomarkers have recently been explored in PD research (Table 2). 

Several studies have sought to classify gene expression profiles in PD for diagnostic purposes [23,24], and three forms of non-coding RNAs have been investigated as potential biomarkers for PD: microRNA (miRNA), long non-coding RNA (lncRNA) and circular RNA (circRNA) [25,26,27,28].

miRNAs are short RNA molecules that are easily detected in body fluids such as blood, CSF, or saliva. Many studies have compared expression levels of various miRNAs between PD patients and healthy controls [25,26]. For example, Cressatti et al., found that salivary miRNA-153 and miRNA-223 may be able to distinguish PD patients from controls with an area under the curve of 79% (95% confidence interval (CI), 64.5–99.2) and 74% (95% CI, 59.6–93.0), respectively [29]. Similarly, Ravanidis and colleagues identified six circRNAs that may be deregulated in PD patients [30]. The authors combined four of these into one biomarker, which in the same patients had a sensitivity of 75.3%, a specificity of 78%, and an area under the curve of 0.84. It has been suggested that biomarkers should achieve areas under the curve >80% in order to be clinically useful [31]. While current miRNA studies are encouraging, the diagnostic accuracy of a biomarker must be measured in a cohort that is independent of the discovery population.

Furthermore, RNA-based biomarker studies in PD have focused on discerning PD patients with motor symptoms from healthy controls. In the clinic, the difficulty often lies in distinguishing idiopathic PD from other causes of parkinsonian symptoms such as progressive supranuclear palsy, multiple system atrophy, or monogenic PD. In this vein, a recent miRNA study identified dysregulated miRNAs that differed between patients with idiopathic vs. monogenic forms of PD, and they found some overlap between patients carrying *SNCA* and *GBA* mutations [32]. From a diagnostic point of view, a biomarker distinguishing monogenic and sporadic PD could help identify cases caused by de novo mutations.

Establishing reproducible, robust RNA-based biomarkers for PD has been a great challenge, in part because most studies have very small sample sizes and the techniques used to detect and analyze miRNA levels are not standardized [25,26,33]. A recent review found that the sensitivity among 24 miRNA studies looking to distinguish between PD cases and healthy controls ranged from 56.7% to 96%, and their specificity from 63.3 to 92% [26]. As such, thorough replication studies will be crucial before these biomarkers can be considered in the clinic.

An early diagnostic test or a progression biomarker would allow pre-symptomatic or high-risk individuals to make more informed plans for their future and, thus, improve quality of life [34]. Such tools would also enable clinical-phase research to target pathogenic processes at an early stage. The underlying disease-causing process of PD is thought to occur up to two decades before motor symptom onset, suggesting that there is indeed a pathological process to be detected early on [35,36]. Longitudinal, population-based biomarker studies will therefore be crucial for establishing clinically effective biomarkers in PD.

## 3. Alzheimer’s Disease

Alzheimer’s disease (AD) is the most common cause of dementia worldwide and is the most prevalent complex neurodegenerative disease with an estimated 14 million cases in the United States [37] and 115 million globally [38] by the year 2050. AD is characterized clinically by impaired short-term memory coupled with progressive cognitive decline and behavioral changes. AD has two distinct neuropathologies: extracellular β-amyloid protein plaque depositions and intracellular neurofibrillary tangles of hyperphosphorylated tau, resulting in neuronal loss in the cortex.

### 3.1. Genetic Biomarkers

#### 3.1.1. Rare Mutations

Early-onset familial AD cases, classified as individuals with familial AD who exhibit symptoms before the age of 60, make up <1% of cases and are caused by mutations in three genes: the β-amyloid precursor protein (*APP*), presenilin-1 (*PSEN1*), and presenilin-2 (*PSEN2*) [39]. The majority of mutations in these three genes result in autosomal dominant forms of early-onset AD [40]. *APP*, found on chromosome 21, encodes for the amyloid-β precursor protein. *APP* is at the center of the amyloid cascade hypothesis, the theory suggesting that a key event in AD pathology is the deposition of the β-amyloid peptide. Alternative splices of the *APP* gene generate different *APP* proteins, and these aggregated plaques initiate various kinase sequences which ultimately lead to hyperphosphorylated tau and the creation of neurofibrillary tangles [41].

The *PSEN1* and *PSEN2* genes, on chromosomes 14 and 1, respectively, encode presenilins which make up the catalytic subunit of the γ-secretase complex, the complex responsible for the cleavage of amyloid precursor proteins which lead to formation of β-amyloid peptides [42]. Causative mutations in these genes likely either increase overall production of β-amyloid (such as *APP* duplications) or produce β-amyloid peptides more prone to aggregation [40].

The triggering receptor expressed on myeloid cells-2 (*TREM2*) gene on chromosome 6, a transmembrane receptor of the immunoglobulin superfamily, has been identified as an immune signaling hub that activates robust immune remodeling in response to tissue damage. The *TREM2* pathway is essential in restricting the spread of tissue damage [43], and rare mutations in *TREM2* affect amyloid and tau pathologies, implicate the role of microglia in the pathogenesis of AD, and strongly increase the risk for developing AD [44].

#### 3.1.2. Common Variants and Polygenic Risk Scores

Sporadic AD cases make up most diagnoses and affect individuals older than 60 years with no discernible pattern of inheritance, indicating a cumulative effect of common, rare, and environmental contributions for AD risk [39]. Found on chromosome 19, the apolipoprotein E (*APOE*) gene is a core component of production, conversion, and clearance of plasma lipoproteins. *APOE* has three common alleles (ε2, ε3, and ε4), and having the ε4 allele is the commonest genetic risk factor for AD [45]. The ε4 allele increases risk in developing AD at earlier ages and in a dose-dependent manner, where one copy is a threefold increase in risk and two copies of the ε4 allele puts individuals at a 10-fold increased risk, with over 60% of AD cases having at least one ε4 allele [45]. In a meta-analysis conducted by Farrer and colleagues, the ε4 allele was found to be a major risk factor across all ethnicities studied (Caucasian, African-American, Hispanic, and Japanese), between the ages of 40 to 90, and in males and females [46]. While the *APOE* ε4 allele is the strongest genetic risk factor, and accounts for up to 25% of heritability in AD [47], the ε2 allele is the strongest genetic protective factor in AD [48]. However, having the *APOE* ε2/ε2 genotype has been associated with severe pathology in primary tauopathies such as progressive supranuclear palsy and corticobasal degeneration [49].

The most recent GWAS meta-analysis to investigate the genetic etiology of AD was conducted by Bellenguez and colleagues and led to the identification of 42 new loci, totaling 75 known risk associations that were replicated in a separate cohort. PRS based on all 75 known AD loci, totaling 83 variants, was significantly associated with progression of all causes of dementia progression (HR = 1.05 per average risk variant, 95%CI (1.03–1.06), *P* = 1.2 × 10^−13^) [50]. The genes prioritized at these loci were associated with known AD pathways such as amyloid and tau metabolism, endocytosis, and innate immunity. New candidate genes identified in AD were previously found to be associated with other neurodegenerative diseases like *IDUA* in PD, progranulin and *TMEM106B* in frontotemporal dementia (FTD). When a locus is associated with two traits, a colocalization analysis can be used to determine if one variant affects both traits, or if there are two causal variants close to each other [51]. A colocalization analysis showed that the AD risk variant near *IDUA* is likely separate from the PD signal, but the variants in progranulin and *TMEM106B* are likely to contribute risk for both AD and FTD [52].

Despite advances in investigating genetic contributions of AD, the translational impact of these findings to the clinic and as diagnostic measures still remains limited [47]. While it has been demonstrated that PRS is useful in estimating individualized AD risk, a recent multi-center longitudinal analysis by Daunt and colleagues have highlighted that PRS is a simple, effective way of identifying mild cognitively impaired patients who are most likely to decline cognitively due to AD over the span of four years with an area under the curve of up to 79% [53]. PRS, since directly derived from GWAS data, has the inherent limitation that it is based on people of European ancestry. Further research will need to be done to assess the predictive accuracy of PRS in other populations, and without robust accuracy and replication, PRS has not reached the rigorous threshold to be used as a diagnostic tool in the clinic.

### 3.2. Transcriptomic Biomarkers

Finding connections between AD associated genes and pathological mechanisms has been an area of interest to identify robust transcriptomic biomarkers for the diagnosis and progression of AD (Table 2) [47]. In addition to increasing diagnostic accuracy when supplemented with clinical findings and informing pre-symptomatic or high-risk individuals earlier in the disease course, reliable and accurate biomarkers of diagnosis and progression would allow therapeutic targeting at an earlier stage of the disease. Previously, most work in AD transcriptomic biomarkers have focused on amyloid and tau-related biomarkers [54].

Biomarkers in the CSF are preferred over most other biochemical biomarkers because the CSF is isolated from the peripheral system by the blood-CSF barrier and interacts directly with the brain in a bidirectional manner. The three established CSF biomarkers based on the core pathological proteins are the 42-amino acid form of the β-amyloid protein (Aβ_42_), total tau (T-tau), and phosphorylated tau at threonine 181 (P-tau_181_), which have been used diagnostically to validate AD diagnosis in ambiguous clinical dementia diagnosis cases, atypical presentations, and patients with mixed pathologies [55]. The Aβ_42_ biomarker measures β-amyloid, a core component of the amyloid plaques found in the brain due to misfolding of the peptides and is low in the CSF when the individual has AD [56]. Tau protein, generated by the microtubule-associated protein tau (*MAPT*) gene on chromosome 17, is predominantly expressed in neurons and stabilizes internal microtubules. In AD, tau dysfunction leads to tau buildup, and the tau levels are high in the CSF [57]. P-tau_181_ levels in the CSF are high in individuals with AD, though recent work by Janelidze and colleagues postulate that CSF P-tau_217_ outperforms P-tau_181_ and distinguishes dementia from AD versus non-AD dementia better than P-tau_181_ [58].

Blood-based biomarkers are minimally invasive, and therefore favored over CSF biomarkers in terms of scalability and cost-effectiveness [54]. Measures of plasma have been associated with β-amyloid deposition, astrogliosis, and neurodegeneration. A recent observational study conducted by Simrén and colleagues investigated both the diagnostic and prognostic capabilities of the following plasma biomarkers of AD pathology: plasma total β-amyloid (Aβ), the 40- and 42-amino acid forms of β-amyloid (Aβ_42_/Aβ_40_) ratio, T-tau, P-tau_181_, axonal injury (neurofilament light; NfL), and astrogliosis (glial fibrillary acidic protein; GFAp) [59]. Both P-tau_181_ and NfL were increased in individuals with mild cognitive impairment. However, P-tau_181_ was found in higher levels in those initially diagnosed with mild cognitive impairment and later converted to AD than those who did not convert to an AD diagnosis. P-tau_181_ also significantly outperformed the other plasma biomarkers when detecting AD at mild cognitive impairment and dementia stages, with longitudinal analyses indicating higher amounts of P-tau_181_ resulted in faster rates of cognitive decline [59]. In a separate study done by Janelidze and colleagues, plasma P-tau_181_ and CSF P-tau_181_ correlated only in individuals who were Aβ+, stipulating that P-tau_181_ in the CSF and blood may be regulated depending on Aβ status [60].

miRNAs are small non-coding RNA species, about 22 nucleotides long, that work to regulate gene expression by binding to complementary target messenger RNA (mRNA) sequences post-transcriptionally. In addition to miRNAs being expressed in the central nervous system, an estimated 200 mRNAs can be regulated by a singular miRNA, suggesting that dysregulation of miRNA expression is likely associated with multiple diseases [61]. Depending on the upregulation or downregulation of certain miRNAs, common different pathological processes such as Aβ accumulation, synaptic dysfunction, memory dysfunction, toxicity due to tau accumulation, cell death, and inflammation are affected [62,63]. An area of particular interest are miRNAs that manipulate the expression of *APP* or the genes that code for its processing enzymes (α-secretase, β-secretase, and γ-secretase), and how those miRNAs slow down or speed up Aβ accumulation [64,65,66,67]. Another area of interest are miRNAs that regulate *MAPT* affecting tau accumulation and relevant protein kinases affecting tau phosphorylation [68,69], looking at both of the distinct neuropathologies associated with AD. In-depth reviews of other miRNAs currently associated with AD have been expanded on elsewhere [61,70,71], however, further studies will be required to assess if a panel of miRNA biomarkers is sufficient to be clinically and diagnostically useful in the identification of AD patients, discerning AD patients from other neurodegenerative disease, or to monitor the progression of AD.

While similar protein deposits are made in both the CSF and blood, further investigation is required to assess the validity and accuracy of blood-based biomarkers [72]. There is a crucial need for robust blood-based biomarkers to screen for AD risk in large numbers in young and healthy individuals and to start treatment early on in the disease course for pre-symptomatic individuals, as it is a safer, less invasive, and cheaper option than CSF biomarkers [73]. Additionally, recent efforts have been made to identify non-tau and non-Aβ biomarkers for use of monitoring response to treatment in drug trials, such as the therapeutic trials targeting Aβ [74]. From a diagnostic and prognostic perspective, population-based longitudinal studies to identify robust blood-based biomarkers alongside having non-tau and non-Aβ biomarkers could aid in the identification of pre-symptomatic individuals and test the efficacy of therapeutic interventions, respectively.

## 4. Amyotrophic Lateral Sclerosis

Amyotrophic lateral sclerosis (ALS) is a form of motor neuron disease, and it is a fatal neurodegenerative condition characterized by the progressive deterioration of motor neurons in the brain and spinal cord, leading to muscle weakness, atrophy, and death within a few years of disease onset [75]. The global incidence of ALS is currently estimated at ~1.59 cases per 100,000 people per year, with estimates rising steadily over the past few decades [76]. ALS is a highly heterogeneous disease both in terms of its clinical presentation and genetic etiology. There are two recognized forms of ALS: familial (fALS), which accounts for ~10% of cases and is often caused by autosomal dominant inheritance; and sporadic (sALS), which accounts for the remaining cases [77]. In around 10–15% of ALS cases, non-motor defects such as behavioral or cognitive impairment manifest in a way that meets the diagnostic criteria for frontotemporal dementia (FTD), and the two disorders are often described as being the opposite ends of a single syndromic spectrum [77].

Although numerous cellular processes have been implicated in ALS—including membrane trafficking, excitotoxicity, signal transduction, nucleocytoplasmic transport, and neuron projection morphogenesis, among others [78,79,80], the mechanisms that underlie the disease are uncertain. As a result, there has been less progress made towards the diagnosis and treatment of ALS compared to other neurodegenerative diseases. ALS diagnosis is currently based on clinical symptoms and electrophysiological criteria, usually over 12 months after symptom onset accompanying substantial motor neuron degeneration [81,82,83]. There has also been interesting progress in the use of non-invasive brain stimulation as a diagnostic tool for ALS [84]. Current ALS treatments have limited effects on disease survival and progression [85,86] and could greatly benefit from biomarkers that aid in presymptomatic diagnosis, monitoring of disease progression, or stratification of ALS patients for clinical trials. There are currently no reliable biomarkers for the majority of ALS cases, though numerous candidate biomarkers are under investigation.

### 4.1. Genetic Biomarkers

Over the past few decades, over 30 genes have been linked to ALS. Although current genetic testing panels can identify many monogenic forms of disease, known ALS-linked genes only account for about two-thirds of all fALS cases and 10–15% of sALS cases [87]. The remaining 80% of ALS cases have no known monogenic cause [88].

#### 4.1.1. Rare Mutations

The most common genetic causes of ALS are pathogenic rare missense mutations in the superoxide dismutase 1 (*SOD1*), fused in sarcoma (*FUS*), and TAR DNA-binding protein 43 (*TARDBP*) genes, as well as hexanucleotide repeat expansions in the chromosome 9 open reading frame 72 (*C9ORF72*) gene. Mutations in these four genes can lead to the accumulation of cytoplasmic protein aggregates and have been extensively studied as potential biomarkers. Novel ALS-linked genes such as *KIF5A* have also been recently identified [89], though pathogenic mutations in the remaining ALS-linked genes are relatively uncommon in comparison.

*SOD1* was the first gene to be linked to ALS, accounting for ~15 to 30% of fALS cases and ~1% of sALS cases depending on the population [90]. Wild-type SOD1 dimers are involved in critical antioxidant defense mechanisms, but pathogenic mutations in the gene have been suggested to confer a toxic gain of function that results in motor neuron damage. There are currently over 185 mutations throughout the *SOD1* gene that have been associated with ALS, some of which cause more aggressive forms of disease (e.g., A4V, H43R, L84V, G85R N86S, and G93A) and others that lead to slower disease progression (e.g., G93C, D90A, and H46R) [91]. Genetic screening for known *SOD1* mutations could be beneficial not only for ALS diagnosis and predicting disease progression, but also for enrollment in clinical trials of SOD1-targeted therapeutics.

TDP-43, encoded by the TAR DNA-binding protein (*TARDBP*) gene, is the main component of ubiquitinated aggregates present in >95% of all sALS cases, ~50% of FTD cases and numerous other neurodegenerative diseases [92]. Although TDP-43 is a nucleoprotein primarily involved in RNA processing and transcriptional regulation, stress and/or mutation can cause it to relocate to the cytoplasm where it is hyperphosphorylated and truncated at the C-terminus, making it prone to aggregation. Since 2008, at least 48 pathogenic mutations in *TARDBP* have been linked to ALS, primarily clustered in the C-terminal domain [93]. These mutations account for ~1 to 4% of fALS cases [90] and could aid in presymptomatic diagnosis for some ALS patients.

Similar to TDP-43, FUS is a nucleoprotein that is involved in transcriptional regulation through RNA/DNA binding and RNA splicing. Though the two proteins share many cellular roles, FUS also functions in DNA repair mechanisms and regulates distinct RNA targets. Mutations in *FUS* cause ~3 to 6% of fALS cases and <1% of sALS cases [90], and carriers of pathogenic *FUS* and *SOD1* mutations rarely exhibit TDP-43 pathology. There are currently over 50 *FUS* mutations that are associated with ALS/FTD, most of which are missense mutations in the nuclear localization signal (NLS) domain that have been shown to cause the mislocalization of FUS to the cytoplasm. Mutations in the low complexity, prion-like N-terminal domain have also been linked to ALS and similarly cause pathological FUS aggregation [94]. Although most FUS-ALS cases present with autosomal-dominant, early-onset or juvenile forms of disease with rapid progression, some present with slower progressing late-onset ALS, indicating that there could be distinct pathological mechanisms associated with specific *FUS* mutations [94]. *FUS* mutations could therefore be important prognostic biomarkers of fALS and could have implications for personalized treatment.

The intronic hexanucleotide repeat expansion (GGGGCC) in the *C9orf72* gene is the most common genetic cause of both ALS and FTD, affecting ~34% of fALS and ~5% of sALS cases in Europeans and ~2% of fALS and <1% of sALS cases in Asians [90]. While the G4C2 repeat typically ranges from ~5 to 10 copies in healthy individuals, it can range in the hundreds or thousands of copies in ALS patients who carry this expansion [95]. There are two main mechanisms that are thought to underlie C9orf72-ALS. First, a portion of the RNAs that are transcribed from the expanded transcript are subject to non-ATG-mediated translation (RAN translation), resulting in the production of abnormal dipeptide repeat proteins that form neuronal inclusions [96,97]. Second, the expanded transcripts adopt unusual secondary structures known as RNA foci which induce cellular toxicity by sequestering RNA-binding proteins, leading to general RNA misprocessing [98]. Since ALS patients harboring C9orf72 repeat expansions often present with cognitive/behavioral impairment [99], and C9orf72-targeted antisense oligonucleotides are currently under investigation (NCT03626012), genotyping to determine the length of the C9orf72 repeat expansion could be an important biomarker for ALS diagnosis and prognosis, and an inclusion criterion for some clinical trials.

#### 4.1.2. Common Variants

In addition to the genetic variants that are thought to cause ALS, genetic modifiers of ALS risk and progression have also been identified. The largest GWAS meta-analysis to date recently identified a total of 15 loci conferring risk for ALS prioritizing genes through different methodological approaches [88]. Additionally, several studies have explored how genetics influences survival in ALS patients. For instance, loss of function mutations in the ephrin receptor *EPHA4* (e.g., R571Q, and R514X) are associated with longer survival in ALS patients [100], the V249I mutation in the chemokine receptor *CX3CR1* is associated with reduced survival in both ALS [101] and AD [102], and common variants in the *UNC13A* gene are associated with increased ALS susceptibility and reduced survival [103,104]. Variants in numerous other genes, including several that are implicated in familial ALS such as the well-studied *SOD1* variants, have also been proposed to modify ALS risk or phenotype [91,105] and could provide valuable information for patient prognosis. Due to the genetic architecture of this devastating disease, in which only few independent loci have been associated with disease through common variation, PRS studies have been scarce [78].

### 4.2. Transcriptomic Biomarkers

Given the molecular heterogeneity associated with ALS and the central role of RNA processing and dysregulation, considerable effort has been made to understand how changes in RNA transcription disrupt disease-relevant pathways and exacerbate disease effects (Table 2). The use of high throughput technologies such as RNA sequencing and microarray platforms has uncovered numerous mRNAs and miRNAs that are differentially expressed in ALS patients.

Although many studies have investigated the transcriptional changes associated with ALS, there are currently no reliable, ALS-specific mRNA biomarkers, as discussed in a recent systematic review by Vijayakumar et al. [106]. However, some mRNAs are differentially expressed in ALS patients and could potentially serve as diagnostic biomarkers. These include kinesins (e.g., KIF5C and KIFC3) and the dynactin subunit DCTN1 which are involved in axonal transport [107,108,109], neurotrophic factors (e.g., Trk-B, BDNF, PI3K, AKT, NFκB, GSK3β, and FASL) involved in cell proliferation and differentiation [110], apoptotic regulatory proteins CyFIP2 and RbBP9 [111], the vascular endothelial growth factor-A (VEGF-A) and chemokine ligand (CCL2) which are thought to play a role in neuroprotection [112], and the transcription factor Nurr1 which is involved in neuroinflammation [113]. However, most of these mRNAs are similarly dysregulated in other neurodegenerative diseases or have not been tested for ALS specificity, with exceptions for FasL mRNA, which showed a significant increase in the peripheral blood leukocytes (PBL) of ALS patients relative to PD, ataxia, and healthy controls [114], and Nurr1, which was downregulated in the peripheral blood of PD patients [114] and upregulated in ALS [113]. Further, VEGF-A and CCL2 mRNAs showed higher elevation in the PBL of Indian ALS patients with respiratory dysfunction and could therefore be disease progression biomarkers [112]. The expression COL19A1 mRNA, which is involved in maintaining muscle integrity, has also been proposed as a prognostic biomarker of ALS [115]. In addition to these potential biomarker candidates, there has been minimal overlap in the mRNAs that are differentially expressed across studies and further studies are needed to determine if these mRNAs can reliably differentiate ALS from ALS mimic syndromes.

Similar to mRNAs, expression profile studies have found that many miRNAs are dysregulated in ALS patients and have been investigated as diagnostic and prognostic biomarkers. Although the role of miRNAs in ALS pathogenesis is more complex than other neurodegenerative diseases, miRNAs could be better biomarker candidates than mRNAs due to their stability in many human biofluids and potential dysregulation in earlier stages of disease [116]. Despite substantial heterogeneity in the miRNAs that are dysregulated across studies, the downregulation of miR-1234-3p in sALS and miR-1825 in both sALS and fALS has been consistently observed in patient serum and could have high diagnostic value since it is specific to ALS relative to AD and Huntington’s disease [117,118].

In addition, miR-206, which slows ALS progression by promoting skeletal muscle growth and regeneration, has been consistently reported to be upregulated in the serum, plasma and PBL samples of ALS patients [119,120,121,122]. Although the upregulation of miR-206 has been identified in other muscular disorders [123,124], it is a promising prognostic biomarker of already-diagnosed ALS since higher serum expression is associated with slower changes in muscle power in ALS patients over one year [121]. miR-338-3p, which regulates neuromuscular junctions, was also reported to be upregulated in the serum, PBL, CSF and spinal cord from sALS patients compared to healthy controls and Parkinson’s, Alzheimer’s, and Huntington’s disease patients [125,126]. Meanwhile, several miRNAs that are dysregulated in ALS patients are involved in ALS-related pathways, including neurogenesis (e.g., miR-9), apoptosis (e.g., let-7d, miR-125b, and miR-155), muscle regeneration (myo-miRNAs: e.g., miR-1, miR-27a, miR-133a, and miR-133b) and neuroinflammation (e.g., miR-125b, miR-155, and miR-146a) [122,127,128,129,130,131,132]. Given the inconsistent results of ALS miRNA profiling studies, using a panel of miRNAs rather than a single miRNA biomarker could have valuable clinical applications for distinguishing ALS cases from both healthy controls and ALS mimic syndromes.

## 5. Future Directions and Conclusions

In recent decades, progress has been made in the development of biomarkers that can inform clinicians and drug developers of critical molecular mechanisms in the neurodegenerative disease process. Success in the development of biomarkers requires extensive scientific collaboration, and cooperation in an open science environment will accelerate this.

Genetic screenings in patients with early-onset neurodegenerative conditions can confirm and refine diagnosis or predict disease in very specific situations. However, in the vast majority of cases, single genomics or transcriptomics biomarkers cannot operate in isolation. There is an increasing need of generating harmonized data across sites to build well-powered biomarker studies by using deep learning and artificial intelligence to combine different types of markers including neuroimaging, *-omic, clinical, and fluid biomarkers. Imaging-related biomarkers are key and have been widely reviewed elsewhere [133,134].

In this arena, the Parkinson’s Progression Marker Initiative and the Accelerating Medicines Partnership for PD and the various efforts for AD and ALS are promising initiatives that will allow data and researchers to connect and work in concert to build better, more effective biomarker panels.

So far, no single biomarker has achieved sufficient accuracy in isolation to be diagnostic for neurodegeneration. As such, research into a multi-modal combination of genetics, imaging, clinical and/or sensor data (such as accelerometry) may provide a more promising approach compared to the search for a single “silver bullet”. Multi-modal biomarkers could be used to chart patient risk, progression, and disease trajectories. Nevertheless, genetic sequencers, neuroimaging scanners and other sensors are not universally available, limiting the price and accessibility of multi-modal diagnostic approaches.

Some major gaps exist in the neurodegenerative disease biomarker space. In particular, these include longitudinal data relating to progression of disease(s) in diverse populations with well characterized outcomes. Diversity on a genetic and genomic scale will be key for applicability and generalizability of findings, as well as increasing the sheer number of relevant candidate biomarkers through methods such as trans-ethnic fine-mapping. These longitudinal cohorts should also include pre-diagnostic cases and leverage existing biomarker work to identify high risk individuals for follow-up from biobanks, healthcare systems or similar resources. From a drug development perspective, these longitudinal case cohorts of well characterized individuals are important as preventing disease onset is an extremely difficult aim and halting disease progress may be a more attainable goal and more efficient use of resources.

## Figures and Tables

**Table 1:** Genetic biomarkers for neurodegenerative diseases.

Gene	Protein	Neurodegenerative Disease
*SNCA*	Alpha-synuclein	monogenic PD
*LRRK2*	Leucine-Rich Repeat Kinase 2	monogenic PD
*PINK2*	PTEN-induced kinase 1	monogenic PD
*PARK2*	Parkin	monogenic PD
*DJ-1*	DJ-1	monogenic PD
*VPS35*	Vacuolar protein sorting ortholog 35	monogenic PD
*GBA*	Glucocerebrosidase	PD (risk factor)
*APP*	Amyloid precursor protein	monogenic AD
*PSEN1*	Presenilin 1	monogenic AD
*PSEN2*	Presenilin 2	monogenic AD
*APOE (ε4 allele)*	Apolipoprotein-E	AD (risk factor)
*TREM2*	TREM2	AD (risk factor)
*TARDBP*	TDP-43	monogenic ALS
*SOD1*	Superoxide dismutase 1	monogenic ALS
*FUS*	Fused-in sarcoma	monogenic ALS
*C9orf72*	C9orf72	ALS (risk factor)
*KIF5A*	KIF5A	ALS (risk factor)

**Table 2:** Potential transcriptomic biomarkers in neurodegenerative diseases.

Gene	Tissue/Biofluid	Upregulated/Downregulated	Neurodegenerative Disease
*miRNA-153*	Saliva	Downregulated	Sporadic PD
*miRNA-223*	Saliva	Downregulated	Sporadic PD
*MAPK9_circ_0001566*	PBMCs	Downregulated	Sporadic PD
*HOMER1_circ_ 0006916*	PBMCs	Downregulated	Sporadic PD
*SLAIN1_circ_0000497*	PBMCs	Downregulated	Sporadic PD
*DOP1B_circ_0001187*	PBMCs	Downregulated	Sporadic PD
*RESP1_circ_0004368*	PBMCs	Downregulated	Sporadic PD
*PSEN1_circ_0003848*	PBMCs	Downregulated	Sporadic PD
*miR-7-5p*	Plasma	Upregulated	Sporadic PD
*miR-22-3p*	Plasma	Upregulated	Sporadic PD
*miR-124-3p*	Plasma	Upregulated	Sporadic PD
*miR-136-3p*	Plasma	Upregulated	Sporadic PD
*miR-139-5p*	Plasma	Upregulated	Sporadic PD
*miR-330-5p*	Plasma	Upregulated	Sporadic PD
*miR-433-3p*	Plasma	Upregulated	Sporadic PD
*miR-495-3p*	Plasma	Upregulated	Sporadic PD
*APOE*	CNS	Upregulated	Sporadic AD
*TREM2*	CNS	Upregulated	AD
*APP; β-amyloid protein (Aβ42/Aβ40)*	CSF; Blood/Plasma	Upregulated	Familial AD
*MAPT (Phosphorylated tau 181 or 231)*	CSF; Blood/Plasma	Upregulated	Sporadic AD
*MAPT (Total tau)*	CSF; Blood/Plasma	Upregulated	Sporadic AD
*NEFL (NfL; neurofilament light chain)*	CSF; Blood/Plasma	Upregulated	Sporadic AD
*GFAP (Glial fibrillary acidic protein)*	Blood/Plasma	Upregulated	AD
*miR-101*		Downregulated	AD
*miR-153*		Downregulated	AD
*miR-346*		Upregulated	AD
*miR-342-3p*	Blood/Plasma	Upregulated	AD
*miR-455-3p*	CNS; Serum	Upregulated	AD
*miR-146a*	CSF	Upregulated	AD
*miR-34a-5p*	CNS; Serum	Upregulated	AD
*miR-93*	Serum	Downregulated	AD
*miR-127-3p*	CSF	Downregulated	AD
*KIF5C*	CNS, PBMCs	Downregulated	Sporadic ALS
*KIFC3*	CNS, PBMCs	Downregulated	Sporadic ALS
*DCTN1*	CNS, PBMCs	Inconsistent results	Sporadic ALS
*Trk-B*	PBL	Downregulated	ALS (non-specific)
*BDNF*	PBL	Downregulated	ALS (non-specific)
*PI3K*	PBL	Downregulated	ALS (non-specific)
*AKT*	PBL	Downregulated	ALS (non-specific)
*NFκB*	PBL	Downregulated	ALS (non-specific)
*GSK3β*	PBL	Downregulated	ALS (non-specific)
*FASL*	PBL	Upregulated	ALS
*CyFIP2*	hMSC, PBL	Upregulated	Sporadic ALS
*RbBP9*	hMSC, PBL	Upregulated	Sporadic ALS
*VEGF-A*	PBMCs	Upregulated	Sporadic ALS
*CCL2*	PBMCs	Upregulated	Sporadic ALS
*Nurr1*	Whole blood	Upregulated	ALS
*COL19A1*	Whole blood	Upregulated	ALS (prognosis)
*miR-1234-3p*	Serum, Plasma	Downregulated	Sporadic ALS
*miR-1825*	Serum, Plasma	Downregulated	ALS
*miR-206*	Serum, Plasma, PBL	Upregulated	Sporadic ALS (non-specific)
*miR-338-3p*	PBL, Serum, CSF	Upregulated	Sporadic ALS (non-specific)
*miR-9*	Plasma, CSF, PBL	Upregulated	Sporadic ALS (non-specific)

The current table represents some of the examples discussed in the text. It does not include by any means a complete list of the numerous differently expressed genes that have been associated with neurodegenerative conditions in the extensive literature. CNS = central nervous system, PBL = peripheral blood leukocytes, hMSC = human mesenchymal stem cells, PBMC = peripheral blood.

## Data Availability

Not applicable.
